# Synthesis, optical and electrochemical properties of ZnO nanowires/graphene oxide heterostructures

**DOI:** 10.1186/1556-276X-8-133

**Published:** 2013-03-22

**Authors:** Huidan Zeng, Ying Cao, Shufan Xie, Junhe Yang, Zhihong Tang, Xianying Wang, Luyi Sun

**Affiliations:** 1 School of Materials Science and Engineering, University of Shanghai for Science and Technology, Shanghai 200093, China; 2 Key Laboratory for Ultrafine Materials of Ministry of Education, School of Materials Science and Engineering, East China University of Science and Technology, Shanghai 200237, China; 3 Department of Chemistry and Biochemistry & Materials Science, Engineering, and Commercialization Program, Texas State University–San Marcos, San Macros, TX 78666, USA

**Keywords:** ZnO, Nanowires, Graphene oxide, Optical properties, Electrochemical properties

## Abstract

Large-scale vertically aligned ZnO nanowires with high crystal qualities were fabricated on thin graphene oxide films via a low temperature hydrothermal method. Room temperature photoluminescence results show that the ultraviolet emission of nanowires grown on graphene oxide films was greatly enhanced and the defect-related visible emission was suppressed, which can be attributed to the improved crystal quality and possible electron transfer between ZnO and graphene oxide. Electrochemical property measurement results demonstrated that the ZnO nanowires/graphene oxide have large integral area of cyclic voltammetry loop, indicating that such heterostructure is promising for application in supercapacitors.

## Background

ZnO nanowires (NWs) and graphene are two of the most widely studied nanomaterials; both of them are good candidates for the electrode materials of supercapacitors. Due to the extremely large surface areas and the pseudocapacitance rising from charge transfer between the electrolyte and electrode through fast Faraday redox reaction, ZnO NWs are promising in the application of supercapacitors [1,2]. On the other hand, graphene has extremely high electron mobility, excellent rate capability, reversibility, and high chemical stability; it has improved electrochemical performance compared with other carbon family materials such as activated carbon, carbon nanotubes, etc. [3]. Moreover, graphene oxide (GO) is considered to be a better choice for the electrodes of supercapacitors than graphene [4]. However, both ZnO NWs and GO suffered from limitations in the real applications. For ZnO NWs, it exhibits low abundance and exhibit poor rate capability and reversibility during the charge/discharge process. For the GO, it is still limited by the low capacitance. Therefore, it is highly desirable for integrating these two materials together because both the double-layer capacitance of GO and pseudocapacitance of ZnO NWs can contribute to the total capacitive performances. Though a few reports have been found on the electrochemical properties of ZnO nanostructures/GO nanocomposites [5-8], however, research on the performance of vertically aligned ZnO NWs/GO heterostructures are very limited although much progress in the controllable synthesis of vertically aligned ZnO nanorods on GO or graphene has been made [9-12].

In this letter, vertically aligned ZnO NWs were grown on GO films using low-temperature hydrothermal method. The optical properties and electrochemical properties of the ZnO NWs/GO heterostructures were studied. Our results showed that the oxygen-containing groups on the surface of GO films can act as the nucleation sites and facilitate the vertical growth of ZnO NWs. Photoluminescence (PL) spectra demonstrated that the deep-level light emission of ZnO NWs grown on GO films were greatly suppressed. Electrochemical property measurement proved that the capacitance of the ZnO NWs/GO heterostructures were much larger than that of the single GO films or ZnO NWs, indicating that such a structure can indeed improve the performance of supercapacitors. Since ZnO NWs are widely studied as sensors, nanogenerators, etc. [13-15] and reduced GO is a good transparent electrode material, we believe that such ZnO NWs/GO heterostructures presented here will also have many other potential applications in all kinds of nanodevices.

## Methods

**Figure 1:**
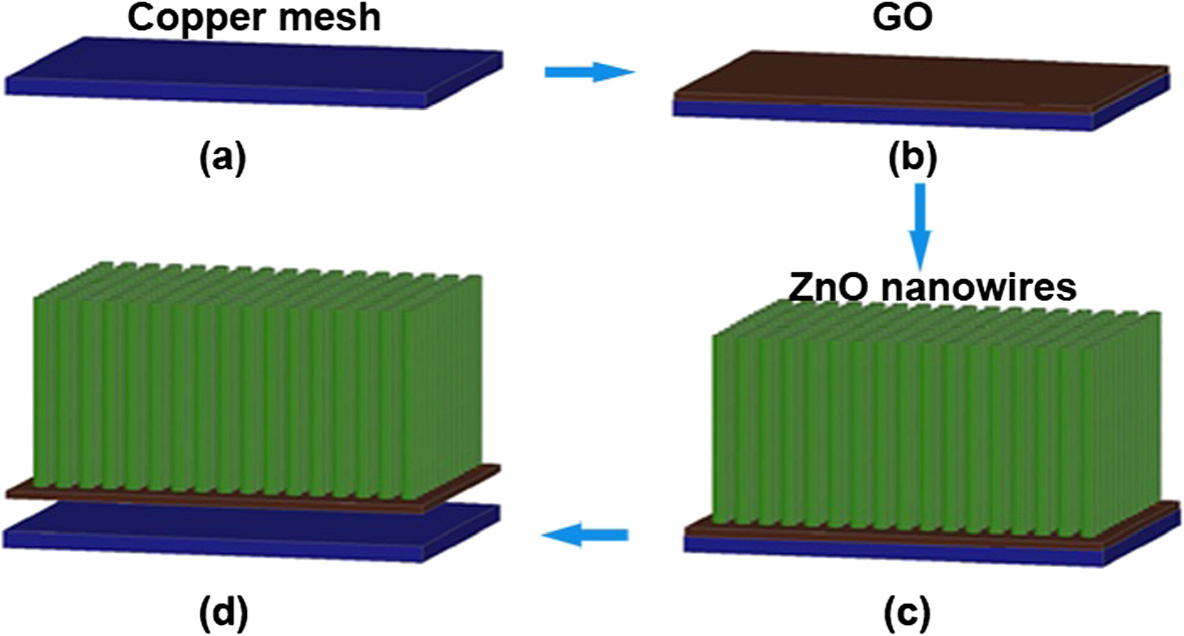
Schematic diagram of the fabrication process of ZnO NWs/GO heterostructures.

Overall, the procedures to synthesize ZnO NWs/GO heterostructures are as follows (Figure 1): (a) pretreating a copper mesh using an ultrasonic cleaner, (b) coating GO film onto the copper mesh substrate, (c) hydrothermal growth of ZnO NWs, and (d) separating the copper mesh from the ZnO NWs/GO heterostructure.

GO film was synthesized via a modified Hummers method. The product was dispersed in deionized water by a Branson Digital Sonifier (S450D, 200W, 40%; Branson Ultrasonics Corporation, Danbury, CT, USA). A dialysis process was used to completely remove residual salts and acids. The purified GO were then dispersed in deionized water to form a homogenous suspension (weight percent: 0.05 wt.%). Subsequently, the GO suspension was drop-casted on the clean copper mesh. After drying, the GO films was used as the substrate for the subsequent hydrothermal growth of ZnO NWs. Equimolar solutions of hexamethylenetetramine (99.9%, Sigma-Aldrich, St. Louis, MO, USA) and zinc nitrate (Zn (NO_3_)_2_ · 6H_2_O) (99.9%, Sigma-Aldrich, St. Louis, MO, USA) were mixed thoroughly and transferred to polymer autoclaves to serve as the precursors. The hydrothermal reaction was carried out at 90°C for 6 h for growing ZnO NWs. After NW growth, the substrate was cleaned with deionized water and then dried at 60°C for 1 h. Finally, the ZnO NWs/GO heterostructure was peeled off from the copper mesh for characterization.

The microstructures of ZnO NWs were characterized by transmission electron microscopy (TEM, Tecnai G2, FEI, Hillsboro, OR, USA), X-ray diffraction (XRD, D8-ADVANCE, Bruker AXS, Inc., Madison, WI, USA) with 0.154 nm Cu Kα radiation, and Raman spectroscopy (laser wavelength 514 nm, via Reflex spectrometer, Renishaw, Wotton-under-Edge, UK). The morphologies of ZnO NWs were examined using a scanning electron microscope (SEM, Quanta FEG, FEI, Hillsboro, OR, USA). Room temperature PL spectra were obtained with a HORIBA Jobin Yvon Fluorolog-3 fluorescence spectrometer (HORIBA Process and Environmental, Les Ulis, France) with an excitation wavelength of 325 nm. A typical three-electrode experimental cell equipped with a working electrode, a platinum foil counter electrode, and a standard calomel reference electrode was used to measure the electrochemical properties. All electrochemical measurements were carried out in 0.10 M Na_2_SO_4_ electrolyte. The cyclic voltammetry (CV) curves were recorded on a CHI660B electrochemical working station (CH Instruments, Austin, TX, USA).

## Results and discussions

**Figure 2:**
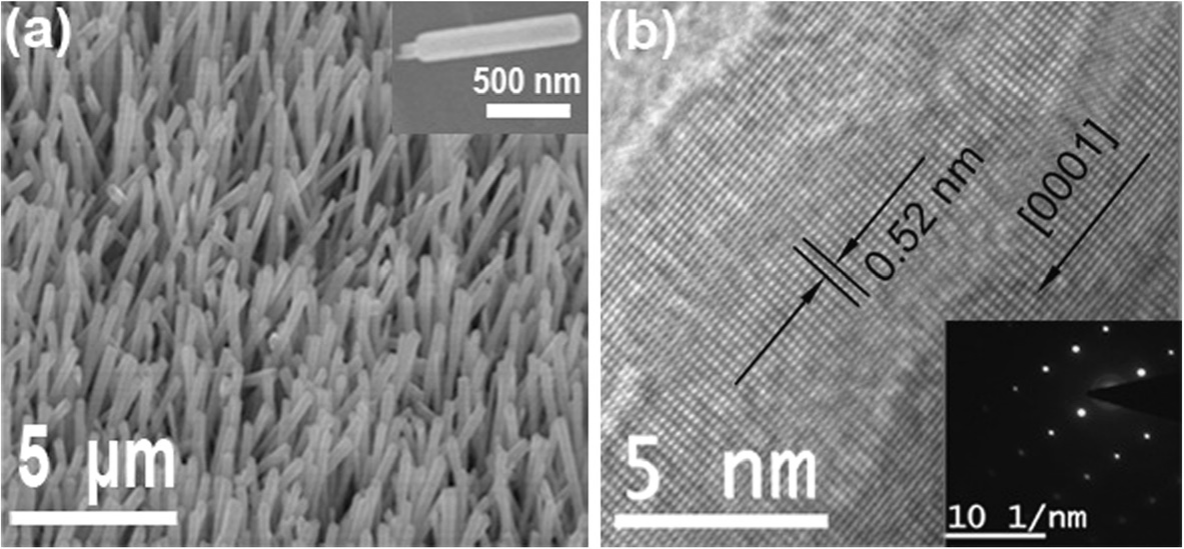
**Characterizations of ZnO NWs.** (**a**) SEM image of ZnO NWs grown on GO film, Inset: high magnification SEM image of a single NW. (**b**) High-resolution TEM image of ZnO NWs. Inset: SAED pattern.

Figure 2 shows the morphologies and microstructures of the ZnO NWs/GO heterostructure. As can be seen from the SEM image of Figure 2a, ZnO NWs are primarily well aligned on GO films, with the diameter ranging from 120 to 180 nm. A high magnification SEM image in the inset of Figure 2a reveals that the root of the NW was anchored to the GO film. The high-resolution TEM image (Figure 2b) confirms the single crystalline structure with a 0.52-nm lattice spacing (i.e., *c*-axis growth direction). The selected area diffraction pattern (SAED) (Inset in Figure 2b) shows that the NW has single crystalline wurtzite structure with growth direction along the <0001> direction.

**Figure 3:**
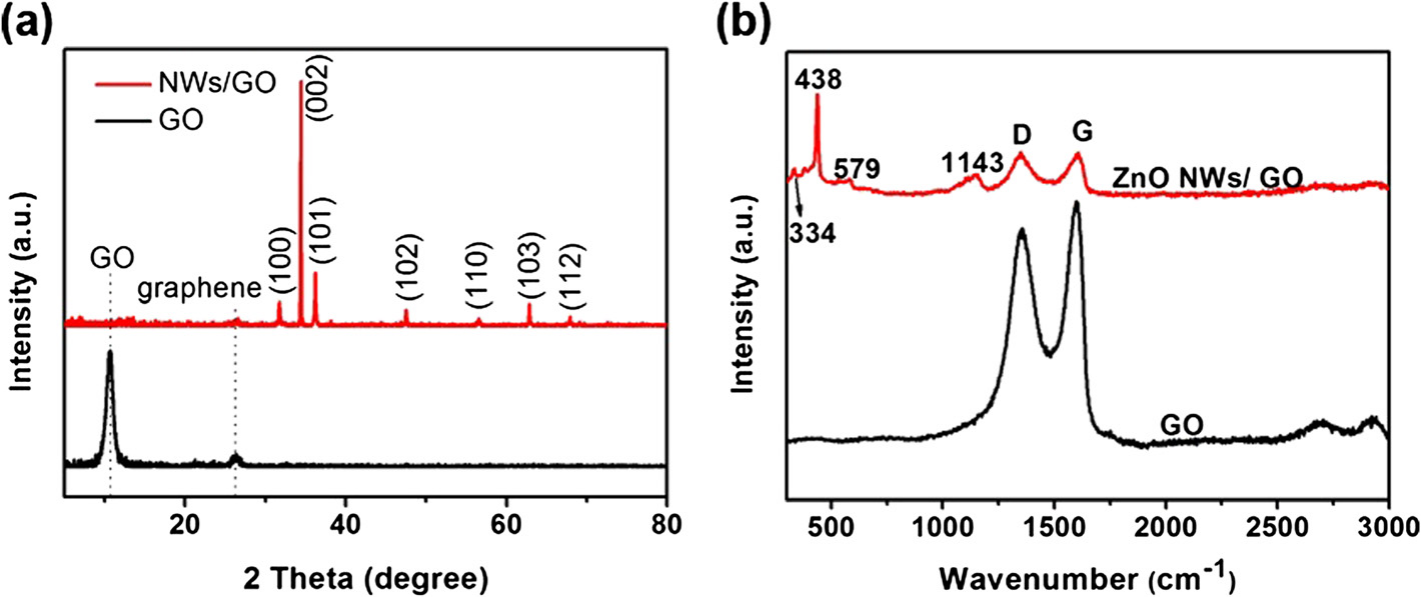
**XRD and Raman spectra.** (**a**) XRD patterns and (**b**) Raman spectra of single GO film and ZnO NWs/GO heterostructures.

Figure 3 shows the XRD and Raman spectra of pure GO film and ZnO NWs/GO heterostructure. Signals related with GO, graphene, and ZnO signals were detected in the XRD patterns. Before the growth of ZnO NWs, a strong and sharp characteristic GO peak at around 10.6° (8.31 Å) was detected, which corresponds to the (*002*) plane of GO films. Meanwhile, a weak (*002*) graphene peak located at 26.4° (3.31 Å) was observed, which indicates that the GO film may contain a tiny concentration of unoxidized graphene. In comparison, after the growth of ZnO NWs, seven peaks located at 2*θ* values of 31.7°, 34.6°, 36.6°, 47.5°, 63°, and 68° can be observed, corresponding to the ZnO crystal planes of (*100*), (*002*), (*101*), (*102*), (*110*), (*103*), and (*112*), respectively. All of these peaks match the wurtzite-structured ZnO. The (*002*) peak of the ZnO NWs/GO heterostructure is much stronger than others, indicating that ZnO NWs have high degree of vertical alignments on the GO film. The GO related peak becomes very weak after the growth of NWs, suggesting that it is fully covered with ZnO NWs.

The Raman spectra of the samples before and after ZnO NW growth are revealed in Figure 3b. Four peaks at 334, 438, 579, and 1143 cm^−1^ are observed in the spectra of ZnO NWs/GO heterostructure. The peak at 438 cm^−1^ corresponds to the finger signal of the characteristic E_2_ mode of ZnO wurtzite structure, while the peaks at 334 and 579 cm^−1^ are attributed to the transversal optical modes with A_1_ symmetry and the longitudinal optical (LO) modes. The peak at 1143 cm^−1^ belongs to the Raman 2LO mode of ZnO.

Two characteristic peaks (D and G bands) of GO can be seen in both curves (Figure 3b). The D-band at 1345 cm^−1^ is due to the A_1g_ mode breathing vibrations of six-membered sp^2^ carbon rings and requires a defect for its activation, and the G-peak at 1598 cm^−1^ corresponds to the E_2g_ vibrational mode of sp^2^ carbon pairs in both rings and chains. In general, the I_D_/I_G_ ratio is a measure of the degree of disorder and average size of the sp^2^ domains in graphene materials^23^: the increased I_D_/I_G_ intensity ratio generally suggests a decrease in the average size of the sp^2^ domains upon the reduction of the GO and the removal of the oxygen functional groups in GO films. The values of I_D_/I_G_ in GO and ZnO NWs/GO heterostructure are calculated to be 0.871 and 1.006, respectively. The increased I_D_/I_G_ ratio in NWs/GO heterostructure suggests that there is a nanostructure change of GO and the average size of the sp^2^ domains decrease. Such structure changes can be attributed to the variation of oxygen functional groups. It was reported that at the initial stage of the reaction, zinc ions are adsorbed on GO films through coordination interactions of the C-O-C and -OH or ion-exchange with H^+^ from carboxyl. In our case, zinc ions are expected to be attached on and reacted with oxygen functional groups to form zinc oxide nuclei, resulting in partial removal of oxygen functional groups from GO films, which is consistent with the observation that the growth of ZnO NWs via hydrothermal method can induce the nanostructure change in GO films.

**Figure 4:**
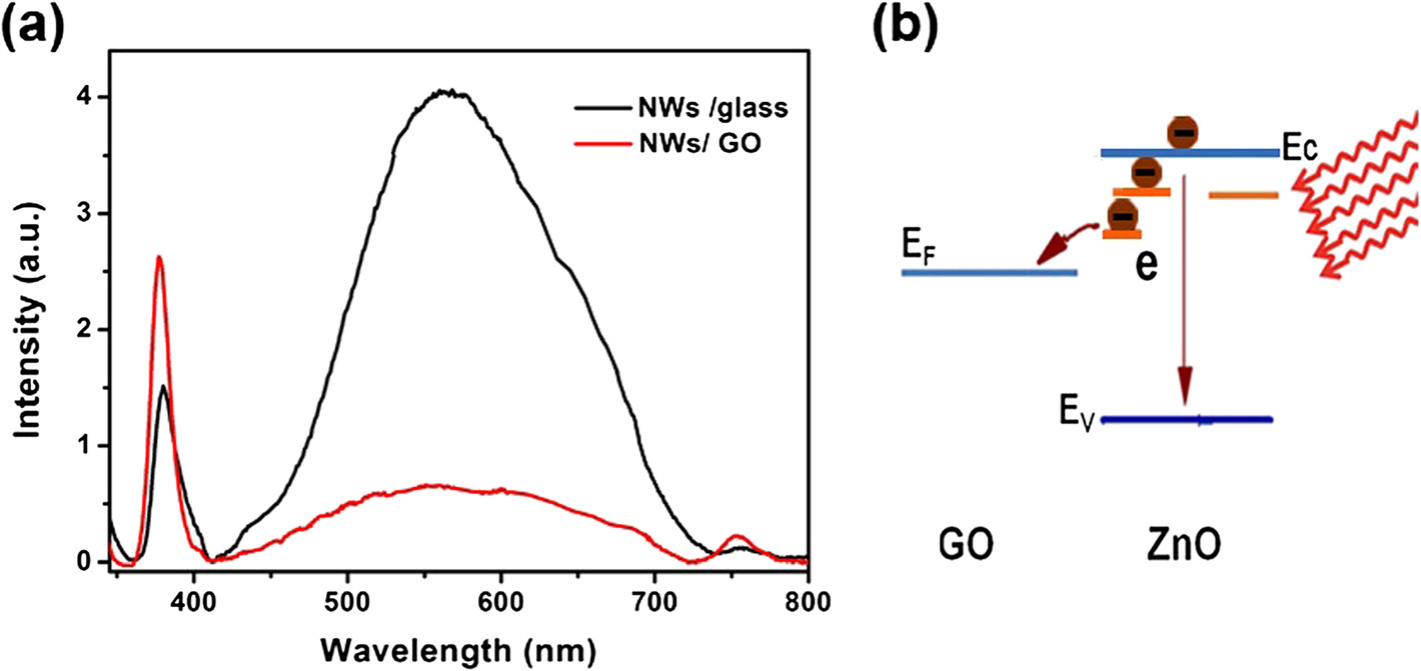
**Comparison of the PL spectra of ZnO NWs grown on GO films and glass substrate.** (**a**) Visible emissions of the ZnO NWs/GO films. (**b**) A schematic diagram of the electron transfer between ZnO NWs and GO films.

Figure 4 shows the PL spectra of ZnO NWs grown on GO films and glass substrates. The samples were fabricated exactly under the same conditions and the growth time was 6 h. For the NWs grown on the glass substrate, the PL spectrum exhibits near-band-edge emission centered at 378 nm and defect emission at around 568 nm. Obviously, the defect-related emission is much stronger than the UV emission, which may be caused by the relatively low crystal quality of hydrothermal grown NWs. In particular, for the NWs grown on the GO films, the near-band UV emission is greatly enhanced and the visible emission of ZnO NWs is greatly suppressed. The relative intensity ratio of these two peaks often has implications on the crystal quality and trapped defect conditions. The intensity ratio of the UV peak and visible peak (*I*_uv_/*I*_vis_) is 4.33, which is much larger than that of the sample grown on glass substrate (0.37). We contribute this effect to the improved crystal quality or the possible electron transfer between ZnO NWs and GO films. The oxygen-containing functional groups on GO films may facilitate the initial nucleation of ZnO NWs and decrease the number of deep-level defects. On the other hand, the visible emission quenching may be caused by the electron transfer between the excited ZnO and GO sheets (Figure 4b). As shown in Figure 4b, under the UV light radiation, some electrons in the conduction band fell back to the valence band and emitted UV light at 378 nm. However, some electrons were trapped in the defect states and transported from ZnO to GO rather than fell back to the ZnO valence band. Therefore, the visible light emission was suppressed. Thus, the visible emissions in Figure 4a are weaker in ZnO NWs/GO films than in bare ZnO NWs.

**Figure 5:**
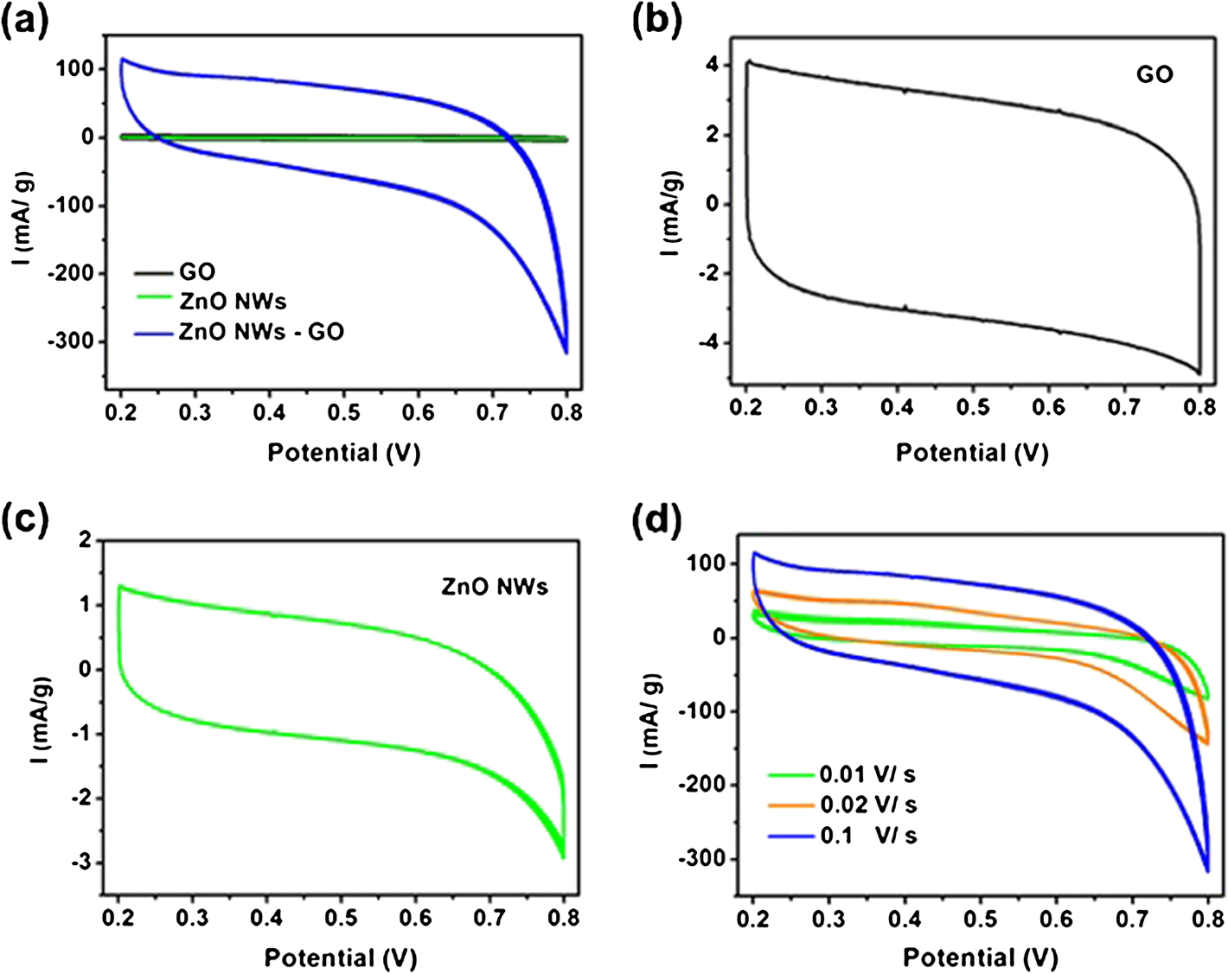
**Electrochemical behavior of the ZnO NWs/GO heterostructures.** (**a**) CV curves of GO, ZnO NWs, ZnO NWs/GO heterostructure. (**b**) Magnified CV curve of GO. (**c**) Magnified CV curve of ZnO NWs. The scan rate of curves in (a-c) is 100 mV s^−1^. (**d**) CV curves of ZnO NWs/GO heterostructure at different scan rates.

In order to illustrate the positive synergistic effect, we characterized the electrochemical performances of the GO films, ZnO NW arrays, and ZnO NWs/GO heterostructures. The CV characterization was performed in 0.1 M NaSO_4_ electrolyte at a scan rate of 100 mV s^−1^. The results (Figure 5a) show that the CV loop of ZnO NWs/GO heterostructure has the largest integral area among the three samples, which indicates that the composite has positive synergistic effects in specific capacitance. This can be attributed to the unique three-dimensional nanostructure of the ZnO NWs/GO. This structure facilitates fast electron transfer between the active materials and the charge collector. In addition, NWs can present as transport channels for more electrical charges to store and transfer in the electrodes. Also, NWs have large specific surface area, which leads to increasing the effective liquid–solid interfacial area and consequently resulting in the efficient utilization of the active material. Therefore, the heterostructure is promising in constructing supercapacitors.

In comparison, the CV curves of GO films and ZnO NWs arrays are shown in Figure 5b,c, respectively. In Figure 5b, the shape of the CV loop of GO films is close to a rectangle, indicating good charge propagation at the electrode surface. In contrast, due to the internal resistance of the composite electrode, the curve shape of the ZnO NWs arrays is distorted (Figure 5c). In addition, the curve shape of ZnO NWs/GO heterostructure is neither a rectangle (Figure 5a). The CV loops result from the superposition of the electric double-layer capacitance and pseudocapacitance due to the reaction between ZnO and electrolyte, which is mainly governed by the intercalation and deintercalation of Na^+^ from electrolyte into ZnO: ZnO + Na^+^ + e^−^ ← → ZnO Na. Figure 5d shows the cyclic CV curves of ZnO NWs/GO films at different sweep rates. The distorted regular shape of the CV curves reveals double-layer capacitive and pseudocapacitance behaviors, which were due to the large internal resistance of the composite and the redox reaction of ZnO, as aforementioned. It can be seen that the CV curves retain a similar shape for the entire sweep. This indicates that the materials have excellent stability, and the electrolyte ions can diffuse into the GO network.

## Conclusions

In summary, ZnO NWs/GO heterostructures have been successfully prepared via a simple solution approach at low temperature. The results showed that the GO layer can facilitate the vertical growth of ZnO NWs and improve their crystal quality. Visible emission quenching was observed in the PL spectra of ZnO NWs/GO heterostructures. The UV emission was greatly enhanced, and the defect-related visible light emission was suppressed. The heterostructures exhibited reversible electrochemical behavior. The combination of the GO and ZnO NWs enabled such composites to possess positive electrochemical behaviors that are promising as electrode material for supercapacitors. In addition, the prepared materials are expected to have potential applications as catalysts, absorbents, and electrodes for other electronic devices.

## Competing interests

The authors declare that they have no competing interests.

## Authors’ contributions

YC, HDZ, and XYW conceived of the study. YC, ZHT, SFX, and XYW carried out the experiments. HDZ, XYW, JHY, and LYS discussed the results and drafted the manuscript. All authors read and approved the final manuscript.

## References

[B1] Kalpana D Omkumar KS Kumar SS Renganathan NG A novel high power symmetric ZnO/carbon aerogel composite electrode for electrochemical supercapacitor Electrochim Acta 2006 8 1309 1315 10.1016/j.electacta.2006.07.032

[B2] Xia XH Tu JP Zhang YQ Wang XL Fan HJ High-quality metal oxide core/shell nanowire arrays on conductive substrates for electrochemical energy storage ACS Nano 2012 8 5531 5538 10.1021/nn301454q 22545560

[B3] Wang Y Shi ZQ Huang Y Ma YF Wang CY Chen MM Chen YS Supercapacitor devices based on graphene materials J Phys Chem C 2009 8 13103 13107 10.1021/jp902214f

[B4] Xu B Yue S Sui Z Zhang X Hou S Cao G Yang Y What is the choice for supercapacitors: graphene or graphene oxide? Energy Environ Sci 2011 8 2826 2830 10.1039/c1ee01198g

[B5] Chen YL Hu ZA Chang YQ Wang HW Zhang ZY Yang YY Wu HY Zinc oxide/reduced graphene oxide composites and electrochemical capacitance enhanced by homogeneous incorporation of reduced graphene oxide sheets in zinc oxide matrix J Phys Chem C 2011 8 5 2563 2571 10.1021/jp109597n

[B6] Lu T Pan L Li H Zhu G Lv T Liu X Sun Z Chen T Chua DH Microwave-assisted synthesis of graphene–ZnO nanocomposite for electrochemical supercapacitors J Alloys Compd 2011 8 18 5488 5490 10.1016/j.jallcom.2011.02.136

[B7] Bai S Shen XP Graphene–inorganic nanocomposites RSC Advances 2012 8 64 98 10.1039/c1ra00260k

[B8] Qu J Luo CQ Cong Q Synthesis of multi-walled carbon Nanotubes/ZnO nanocomposites using absorbent cotton Nano-Micro Lett 2011 8 2 115 120 10.2174/1876402911103020115

[B9] Kim YG Hadiyawarman Yoon R Kim MY Yi GC Liu CL Hydrothermally grown ZnO nanostructures on few-layer graphene sheets Nanotechnology 2011 8 245603 245608 10.1088/0957-4484/22/24/245603 21508449

[B10] Alver U Zhou W Belay AB Krueger R Davis KO Hickman NS Optical and structural properties of ZnO nanorods grown on graphene oxide and reduced graphene oxide film by hydrothermal method Appl Surf Sci 2012 8 3109 3114 10.1016/j.apsusc.2011.11.046

[B11] Chang HX Sun ZH Ho KY Tao XM Yan F Kwok WM Zheng ZJ A highly sensitive ultraviolet sensor based on a facile in situ solution-grown ZnO nanorod/graphene heterostructure Nanoscale 2011 8 258 264 10.1039/c0nr00588f 20976323

[B12] Choi WM Shin KS Lee HS Choi D Kim KH Shin HJ Yoon SM Choi JY Kim SW Selective growth of ZnO nanorods on SiO2/Si substrates using a graphene buffer layer Nano Res 2011 8 5 440 447 10.1007/s12274-011-0100-6

[B13] Wang XY Kim K Wang YM Stadermann M Noy A Hamza AV Yang JH Sirbuly DJ Matrix-assisted energy conversion in nanostructured piezoelectric arrays Nano Lett 2010 8 12 4901 4905 10.1021/nl102863c 21062047

[B14] Zhang YF Geng HJ Zhou ZH Wu J Wang ZM Zhang YZ Li ZL Zhang LY Yang Z Liang H Wang H Development of inorganic solar cells by nanotechnology Nano-Micro Lett 2012 8 2 124 134

[B15] Hu Y Zhang Y Xu C Lin L Snyder RL Wang ZL Self-powered system with wireless data transmission Nano Lett 2011 8 2572 2577 10.1021/nl201505c 21604749

